# Comparative effectiveness of delayed-release dimethyl fumarate versus interferon, glatiramer acetate, teriflunomide, or fingolimod: results from the German NeuroTransData registry

**DOI:** 10.1007/s00415-018-9083-5

**Published:** 2018-10-16

**Authors:** Stefan Braune, Sarah Grimm, Philip van Hövell, Ulrich Freudensprung, Fabio Pellegrini, Robert Hyde, Arnfin Bergmann, NTD Study Group

**Affiliations:** 1 NeuroTransData, Bahnhofstraße 103B, 86633 Neuburg an der Donau, Germany; 2 PwC Digital Services, Birchstraße 160, 8050 Zurich, Switzerland; 3 Biogen International GmbH, Landis Gyr-Straße 3, 6300 Zug, Switzerland

**Keywords:** Comparative effectiveness research, Dimethyl fumarate, Multiple sclerosis, Propensity score matching, Observational data, Registry

## Abstract

**Background:**

Comparative effectiveness (CE) research allows real-world treatment comparisons using outcome measurements important to physicians/patients. This German NeuroTransData registry-based analysis compared delayed-release dimethyl fumarate (DMF) effectiveness with interferons (IFN), glatiramer acetate (GA), teriflunomide (TERI), or fingolimod (FTY) in patients with relapsing–remitting multiple sclerosis (RRMS) using propensity score matching (PSM).

**Methods:**

Data from registry patients aged ≥ 18 years with RRMS, ≥ 1 relapse, and Expanded Disability Status Scale (EDSS) assessment(s) after index therapy initiation underwent 1:1 PSM to match DMF with comparator populations baseline characteristics. Primary outcome measurement was time to first relapse (TTFR). Secondary outcome measurements included annualised relapse rate (ARR), proportion of patients relapse free at 12 and 24 months, time to index therapy discontinuation (TTD), and reasons for discontinuation. Exploratory analyses included time to 3- and 6-month EDSS confirmed disability progression (CDP). Non-pairwise censoring was the primary analysis method; pairwise censoring was the main sensitivity analysis method.

**Findings:**

Post-matched cohorts were well-balanced. By non-pairwise censoring, TTFR and ARR were significantly lower in DMF populations versus matched IFN, GA, and TERI, but there was no evidence of difference between DMF and FTY. TTD was similar between DMF and IFN, GA, and TERI, but significantly shorter versus FTY. Time to CDP generally showed no evidence of difference between DMF and comparator populations. Pairwise censored analysis results confirmed the non-pairwise censoring results.

**Interpretation:**

These results support previous CE studies in demonstrating relative improvement in real-world effectiveness with DMF versus first-line agents IFN, GA, and TERI, and similar effectiveness versus FTY.

**Electronic supplementary material:**

The online version of this article (10.1007/s00415-018-9083-5) contains supplementary material, which is available to authorized users.

## Introduction

Delayed-release dimethyl fumarate (DMF; also known as gastro-resistant DMF) has been shown to be effective for the treatment of relapsing–remitting multiple sclerosis (RRMS) in randomised clinical trials (RCTs), post hoc analyses, and real-world effectiveness studies [1–5]. The CONFIRM study and subsequent post hoc analyses found DMF had greater efficacy in terms of reduction in annualised relapse rate (ARR) versus placebo or glatiramer acetate (GA) [1, 4]. Post hoc analyses of clinical trial data using mixed treatment comparisons or indirect matching-adjusted methods also found DMF was associated with improved efficacy, as mainly assessed by ARR, compared with interferons (IFN), or teriflunomide (TERI), and with similar efficacy compared with fingolimod (FTY) [2, 3, 5].

Randomised clinical trials and other controlled trials are necessary for satisfying regulatory agencies regarding efficacy and safety of new medications, but provide limited information relevant to clinical settings and health policy decision-making [6]. Controlled trials also are costly to perform and have time constraints. As a result, comparative effectiveness (CE) research has emerged as a means of incorporating real-world clinical data, which may include multiple comparators and patients generally excluded by the strict methodology of RCTs. A key objective of CE research methodologies is to reduce the inherent bias associated with treatment selection and decision. In the case of propensity score matching (PSM), potential baseline (i.e. index therapy initiation) and treatment confounders are matched to ensure comparability between treatment groups at baseline, such as those in a retrospective observational study or with patients drawn from a prospectively designed registry. To further improve the reliability of real-world evidence-based data, analysis-censoring procedures need to be implemented and communicated.

Dimethyl fumarate has been compared with a number of alternative treatments using CE research methods, including claims-based analyses [7, 8], a cross-sectional study [9], and a number of PSM analyses based on registries [10–14]. In the PSM analyses performed to date, DMF showed improved clinical effectiveness in terms of reductions in ARR and time to first relapse (TTFR) events versus IFN, GA, and TERI [13, 14], and a similar rate of relapse as FTY [10–12, 14]. To support this growing body of real-world evidence, this CE analysis based on the German NeuroTransData (NTD) multiple sclerosis (MS) registry was conducted to assess real-world CE of DMF compared with IFN, GA, TERI, and FTY in PSM cohorts of patients with RRMS.

## Methods

### German NTD registry

The NTD is a Germany-wide network of physicians founded in 2008 in the fields of neurology and psychiatry. Currently, 78 neurologists in 153 offices work in NTD practices serving about 600,000 outpatients per year. Each practice is certified according to network-specific and ISO 9001 criteria. Compliance with these criteria is audited annually by an external certified audit organisation. The NTD MS registry includes about 25,000 patients with MS. In the database, demographic, clinical history, and clinical variables are captured in real time during an average of 3.7 visits and Expanded Disability Status Scale (EDSS) assessments per year per patient. A unique relapse definition is applied within NTD. Standardised clinical assessments of functional system scores and EDSS calculation are performed by certified raters (http://www.neurostatus.net/). All personnel undergo regular training to ensure quality of data in the database. Both automatic and manually executed queries are implemented to further ensure data quality. All data are pseudonymised and pooled to form the MS registry database. This data acquisition protocol was approved by the ethical committee of the Bavarian Medical Board (Bayerische Landesärztekammer; June 14, 2012). On average, nearly four EDSS assessments per year were obtained per individual patient.

### Study population

Dimethyl fumarate populations were compared with the following first-line treatment populations: IFN, GA, TERI, and FTY all-comer populations. DMF also was compared with a FTY (European) label population, which includes patients who have either highly active disease that has not responded to other disease-modifying therapies (DMTs) or rapidly progressive disease [15, 16].

Inclusion criteria for all treatment comparisons were: RRMS (10th revision of the International Statistical Classification of Diseases and Related Health Problems codes G35.0, G35.9, G35.10, or G35.11), age at least 18 years, and to ensure a minimal follow-up time within the study, a valid EDSS measurement and/or a relapse after index therapy initiation was required. Median follow-up frequency between visits, including EDSS assessment within NTD, was approximately 3 months. The relapse criterion was introduced to ensure that patients with an early relapse after index therapy initiation were not excluded from the analysis population. For treatment comparisons with IFN, GA, or TERI, patients had to be either treatment naive or have received pre-treatment with other first-line therapy (e.g. GA or TERI in the case of IFN). In addition, patients treated with injectable therapies (IFN, GA) required therapy initiation from January 1, 2010 onwards to better reflect the current treatment landscape given that the introduction of oral therapies occurred at this time. For comparisons with the FTY label population, patients had to have been pre-treated with IFN, GA, or TERI, with an on-therapy relapse within the last 12 months (to reflect the European label) and have switched from pre-treatment indicating treatment failure on first-line therapy, with a treatment gap of up to 6 months. For comparisons with the FTY all-comer population, patients were either treatment naive or had switched from pre-treatment with IFN, GA, or TERI, with a treatment gap of up to 6 months.

Exclusion criteria were pre-treatment with a DMT other than those allowed or specified as part of the inclusion criteria. Specifically, this meant pre-treatment with any DMT other than: GA or TERI for comparisons with IFN, IFN or TERI for comparisons with GA, GA or IFN for comparisons with TERI, and GA or TERI for comparisons with FTY all-comer or label populations.

The analysis population included all patients who satisfied the inclusion/exclusion criteria and started index therapy (dosed at least once) with a relapse measurement and/or EDSS measurement post-index therapy initiation. For each index therapy comparison, any single patient could only participate once, but each patient could contribute to more than one index therapy comparison.

### Study outcome measurements

The primary outcome measurement was TTFR. Secondary outcome measurements were ARR, proportion of relapse-free patients at 12 and 24 months, time to index therapy discontinuation (TTD), and reasons for discontinuation. Time to EDSS confirmed disability progression (CDP) at 3 and 6 months was included as an exploratory outcome measurement. CDP events were defined as at least 0.5-point EDSS score increases for patients with baseline EDSS score greater than 5.5, and at least 1.0-point EDSS score increases for patients with baseline EDSS score 0‒5.5.

### PSM and statistical analysis

No formal sample size was pre-calculated because available data already captured within the NTD registry were used. A 1:1 PSM (5:1 greedy matching algorithm [17]) was used to match measured baseline characteristics of DMF populations to comparator populations for each treatment comparison [18, 19]. Propensity scores were calculated using multiple logistic regression with the treatment cohort as the dependent variable and the following confounders at index therapy initiation as independent variables: age, sex, disease duration (from first clinical symptoms to start of index therapy), treatment history (number of previous therapies), baseline EDSS score, and total number of relapses in the past 12 and 24 months (based on actual follow-up period before index therapy initiation). For all comparisons other than with the FTY label population, treatment history was categorised and matched as 0 (treatment naive), 1, 2, 3, and 4+ (representing number of previous DMTs). For the FTY label population, treatment history was categorised as 1, 2, 3, and 4+ because treatment-naive patients were not included. Wilcoxon rank-sum and Chi-square tests were used to compare unmatched baseline characteristics by cohort, whereas in the matched data, Wilcoxon signed-rank and McNemar or Stuart–Maxwell tests for marginal homogeneity were used to compare baseline characteristics for continuous variables and proportions, respectively. Pre- and post-matching balance in baseline covariates were based on standardised mean differences (threshold 0.10), and the C-statistic [20]. The C-statistic is a measure of balance in matched data and ranges from 0.5 to 1.0, with the minimum value indicating the propensity score model is perfectly balanced and has no ability to discriminate between cohorts after matching.

Time to first relapse, TTD, and time to CDP at 3 and 6 months were all analysed using a Cox marginal regression model taking into account the clustered nature of the matched design. For the confirmation of CDP at 3 and 6 months, EDSS scores recorded within 30 days after the onset of a relapse were excluded. Treatment effects were reported as hazard ratios (HRs) together with 95% CIs, and Kaplan–Meier methods were applied to obtain estimates at pre-defined time points. ARR was calculated as total number of relapses divided by total exposure (years), with treatment effect for ARR estimated using a generalised estimating equations (GEE) Poisson regression model. ARRs (95% CIs) for each cohort were presented and treatment effects reported as rate ratios (RRs), along with 95% robust CIs.

Non-pairwise censoring was the primary analysis method for all major outcome measurements. However, pairwise censoring was performed as a sensitivity analysis to account for potential differences between exposure times and to assess the robustness of the results.

### Role of the funding source

Biogen was involved in study design, data analysis, and manuscript preparation. Biogen did not have access to patient-level data.

## Results

### Demographic and baseline disease characteristics

Overall registry patient numbers and flow are shown in Supplementary Fig. 1. Baseline characteristics of post-matched populations showed no evidence of a difference as confirmed by small standardised differences (i.e. <0.1) and C-statistic values of 0.519–0.586 across treatment comparisons (Tables 1, 2). Further, propensity score density distributions for DMF and all comparators after matching showed very good overlap (Supplementary Fig. 2). Characteristics for the unmatched populations are shown in Supplementary Tables 1 and 2. Exposure time (median, 25th quantile, 75th quantile) via non-pairwise censoring in both populations were generally similar for the comparisons of DMF versus IFN, GA, and TERI (Supplementary Table 3). Exposure times were slightly lower in DMF versus FTY populations due to shorter availability of DMF in Germany: median (25th quantile, 75th quantile) exposure times were 16.3 (7.6, 23.7) months for the DMF cohort and 24.1 (8.6, 41.1) months for the FTY cohort. Similarly, the exposure time for the DMF cohort [15.3 (7.0, 22.6) months] was lower than that of the matched FTY label cohort [22.5 (7.6, 34.4) months]. However, this difference in exposure between DMF and FTY highlighted the importance of conducting the sensitivity analysis based on pairwise censoring to ensure the robustness of the results.

**Table 1:** Baseline characteristics of pre-matched and post-matched populations for the IFN, GA, and TERI comparison populations

Baseline factor	DMF versus IFN								DMF versus GA							
	Pre-matched				Post-matched				Pre-matched				Post-matched			
	DMF (*n* = 474)	IFN (*n* = 1385)	*d*	*p* value	DMF (*n* = 439)	IFN (*n* = 439)	*d*	*p* value	DMF (*n* = 700)	GA (*n* = 709)	*d*	*p* value	DMF (*n* = 535)	GA (*n* = 535)	*d*	*p* value
Female	71.3%	71.7%	0.009	0.918	71.1%	74.5%	0.077	0.301	70.4%	75.0%	0.104	0.06	71.8%	71.2%	0.012	0.885
Age (years)	39.9 (10.56)	36.7 (10.49)	− 0.305	0.000	39.1 (10.39)	39.9 (10.87)	0.079	0.358	39.4 (10.79)	37.9 (10.28)	− 0.142	0.007	39.0 (10.74)	38.9 (10.34)	− 0.011	0.932
Median EDSS (Q25, Q75)	1.5 (1, 2.5)	1.5 (0, 2)	− 0.169	0.004	1.5 (1, 2.5)	1.5 (0, 2.25)	− 0.022	0.830	1.5 (1, 2.5)	1.5 (1, 2)	− 0.107	0.076	1.5 (1, 2.5)	1.5 (1, 2.5)	0.003	0.639
Disease duration in months (SD)	87.3 (87.8)	59.0 (80.8)	− 0.335	0.000	81.0 (83.8)	86.8 (99.3)	0.063	0.858	92.7 (91.1)	66.1 (75.3)	− 0.319	0.000	78.0 (80.4)	78.2 (80.6)	0.003	0.963
Prior number of DMTs, %																
0	69.2%	92.2%			74.7%	77.0%			46.9%	70.9%			60.9%	62.2%		
1	30.0%	7.7%	0.61	0.000	24.6%	22.6%	0.058	0.025	52.0%	28.9%	0.512	0.000	38.9%	37.6%	0.027	0.392
2	0.8%	0.1%			0.7%	0.5%			1.1%	0.1%			0.2%	0.2%		
Total number of relapses in the last 12 months, %																
0	66.7%	54.2%			64.2%	66.1%			66.9%	60.5%			63.2%	64.9%		
1	28.3%	38.3%			30.3%	28.5%			26.6%	31.5%			29.3%	28.2%		
2	4.4%	6.8%	0.269	0.000	4.8%	5.0%	0.079	0.682	5.9%	6.2%	0.157	0.070	6.5%	5.8%	0.047	0.882
3	0.2%	0.5%			0.2%	0.0%			0.6%	1.4%			0.7%	0.9%		
4+	0.4%	0.1%			0.5%	0.5%			0.1%	0.4%			0.2%	0.2%		
Total number of relapses in the last 24 months, %																
0	60.8%	50.5%			58.3%	60.8%			57.1%	55.6%			57.2%	58.3%		
1	28.9%	37.8%			30.8%	27.8%			28.9%	30.5%			28.8%	27.7%		
2	8.4%	9.3%	0.212	0.004	8.9%	9.8%	0.082	0.644	10.4%	9.2%	0.083	0.655	9.5%	9.2%	0.035	0.958
3	1.3%	1.7%			1.4%	0.9%			2.6%	3.7%			3.4%	3.7%		
4+	0.6%	0.6%			0.7%	0.7%			1.0%	1.1%			1.1%	1.1%		
C-statistic	0.692				0.539				0.668				0.524			

Baseline factor	DMF versus TERI							
	Pre-matched				Post-matched			
	DMF (*n* = 917)	TERI (*n* = 388)	*d*	*p* value	DMF (*n* = 388)	TER (*n* = 388)	*d*	*p* value
Female	72.7%	66.8%	0.131	0.035	67.8%	66.8%	0.022	0.813
Age (years)	39.8 (10.60)	44.1 (9.67)	0.419	0.000	44.2 (10.29)	44.1 (9.67)	− 0.017	0.621
Median EDSS (Q25, Q75)	1.5 (1, 2.5)	2 (1, 3)	0.075	0.122	2 (1, 3)	2 (1, 3)	− 0.044	0.572
Disease duration in months (SD)	99.2 (89.1)	119.6 (102.1)	0.214	0.001	122.5 (104.1)	119.6 (102.1)	− 0.028	0.730
Prior number of DMTs, %								
0	35.9%	38.4%			36.1%	38.4%		
1	53.9%	53.6%	0.086	0.380	54.9%	53.6%	0.055	0.737
2	10.3%	8.0%			9.0%	8.0%		
Total number of relapses in the last 12 months, %								
0	68.8%	67.0%			69.6%	67.0%		
1	25.0%	28.6%			26.3%	28.6%		
2	5.5%	4.1%	0.119	0.308	4.1%	4.1%	0.090	0.410
3	0.8%	0.3%			‒	0.3%		
4+	‒	‒			‒	‒		
Total number of relapses in the last 24 months, %								
0	58.2%	55.2%			57.7%	55.2%		
1	27.9%	34.0%			33.2%	34.0%		
2	10.3%	8.5%	0.153	0.179	7.5%	8.5%	0.116	0.620
3	2.5%	1.8%			1.5%	1.8%		
4+	1.1%	0.5%			‒	0.5%		
C-statistic	0.635				0.533			

*d* standardised difference, *DMF* delayed-release dimethyl fumarate, *DMT* disease-modifying therapy, *GA* glatiramer acetate, *IFN* interferons, *EDSS* Expanded Disability Status Scale, *TERI* teriflunomide

**Table 2:** Baseline characteristics of pre-matched and post-matched populations for the FTY all-comer and FTY label comparison populations

Baseline factor	DMF versus FTY all-comer								DMF versus FTY label							
	Pre-matched				Post-matched				Pre-matched				Post-matched			
	DMF (*n* = 793)	FTY (*n* = 773)	*d*	*p* value	DMF (*n* = 457)	FTY (*n* = 457)	*d*	*p* value	DMF (*n* = 99)	FTY (*n* = 420)	*d*	*p* value	DMF (*n* = 99)	FTY (*n* = 99)	*d*	*p* value
Female	71.6%	71.4%	0.005	0.969	72.6%	73.5%	0.020	0.824	75.8%	70.0%	0.130	0.311	75.8%	81.8%	0.149	0.345
Age in years (SD)	40.3 (10.78)	39.3 (10.05)	− 0.095	0.044	39.9 (10.95)	40.2 (9.67)	0.029	0.713	37.8 (9.6)	39.1 (10.3)	0.133	0.265	37.8 (9.6)	37.1 (9.6)	− 0.065	0.624
Median EDSS (Q25, Q75)	1.5 (1, 2.5)	2 (1.5, 3.5)	0.406	0.000	2 (1, 3.5)	2 (1, 3)	0.03	0.689	2 (1, 3.25)	2 (1.5, 3.5)	0.266	0.035	2 (1, 3.25)	2 (1.5, 3)	− 0.015	0.870
Mean disease duration in months (SD)	98.2 (93.3)	106.6 (87.6)	0.093	0.001	104.8 (93.4)	108.1 (92.4)	0.036	0.578	95.2 (81.9)	109.6 (84.4)	0.174	0.068	95.2 (81.9)	93.9 (72.8)	− 0.016	0.955
Prior number of DMT, %																
0	41.5%	13.8%			23.0%	22.1%			–	–			–	–		
1	47.5%	71.5%	0.655	0.000	63.7%	64.1%	0.023	0.909	82.8%	83.1%	0.007	1.000	82.8%	76.8%	0.151	0.361
2	10.6%	14.5%			13.1%	13.6%			17.2%	16.9%			17.2%	23.2%		
3	0.4%	0.1%			0.2%	0.2%			‒	‒			‒	‒		
Total number of relapses in the last 12 months, %																
0	69.4%	35.3%			59.1%	59.3%			1.0%	0.2%			1.0%	1.0%		
1	24.8%	38.8%			31.1%	30.6%			72.7%	58.3%			72.7%	66.7%		
2	5.0%	19.0%	0.793	0.000	8.5%	9.2%	0.059	0.715	22.2%	30.5%	0.372	0.041	22.2%	28.3%	0.141	0.450
3	0.5%	5.8%			0.9%	0.4%			3.0%	9.3%			3.0%	3.0%		
4+	0.3%	1.0%			0.4%	0.4%			1.0%	1.7%			1.0%	1.0%		
Total number of relapses in the last 24 months, %																
0	59.3%	29.5%			50.5%	49.5%			48.5%	34.8%			–	–		
1	28.2%	29.4%			29.3%	30.6%			31.3%	33.6%			48.5%	44.4%		
2	8.8%	22.0%	0.776	0.000	14.2%	13.3%	0.045	0.967	13.1%	19.8%	0.317	0.051	31.3%	31.3%	0.106	0.219
3	2.5%	12.3%			3.9%	4.4%			7.1%	11.9%			13.1%	16.2%		
4+	1.1%	6.9%			2.0%	2.2%			–	–			7.1%	8.1%		
C-statistic	0.780				0.519				0.635				0.586			

*d* standardised difference, *DMF* delayed-release dimethyl fumarate, *DMT* disease-modifying therapy, *EDSS* Expanded Disability Status Scale, *FTY* fingolimod

### DMF versus IFN

**Fig. 1:**
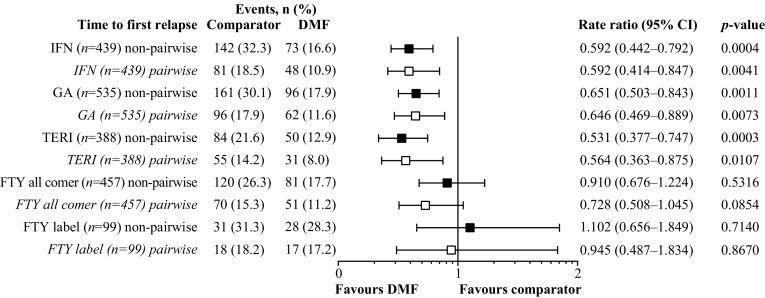
Hazard ratios of time to first relapse for DMF versus comparator populations. *DMF* delayed-release dimethyl fumarate, *FTY* fingolimod, *GA* glatiramer acetate, *IFN* interferons, *TERI* teriflunomide

**Fig. 2:**
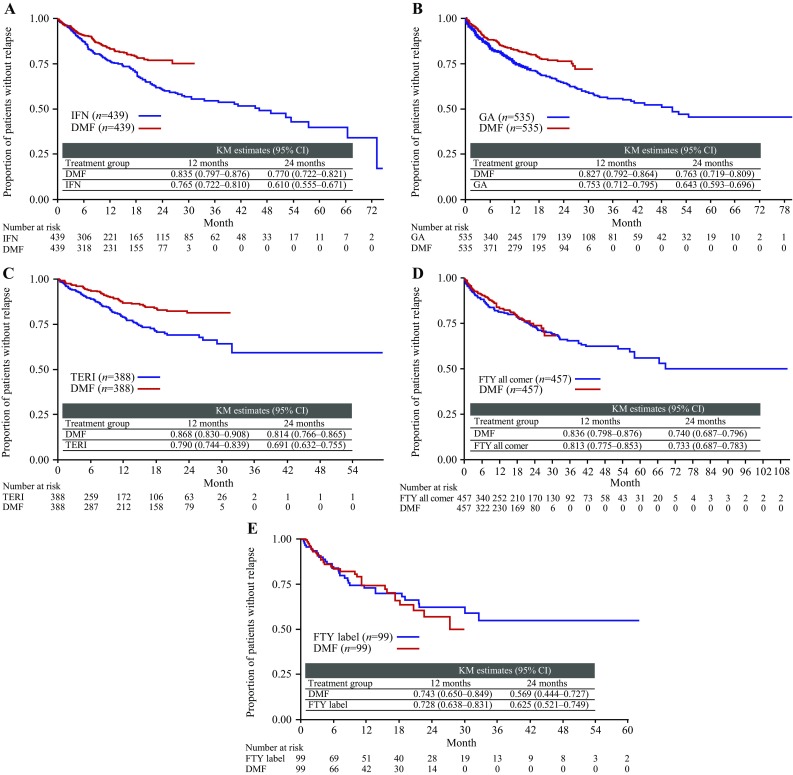
Time to first relapse KM survival curves. **a** DMF versus IFN all-comer population; **b** DMF versus GA all-comer population; **c** DMF versus TERI all-comer population; **d** DMF versus FTY all-comer population, and **e** DMF versus FTY label population. *DMF* delayed-release dimethyl fumarate, *FTY* fingolimod, *GA* glatiramer acetate, *IFN* interferons, *KM* Kaplan–Meier, *TERI* teriflunomide

**Fig. 3:**
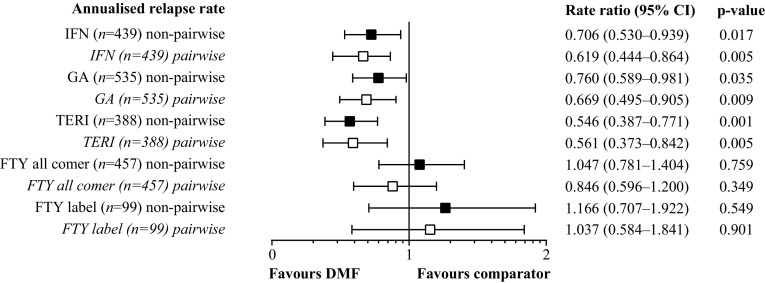
Forest plot of annualised relapse rate for DMF versus comparator populations. *DMF* delayed-release dimethyl fumarate, *FTY* fingolimod, *GA* glatiramer acetate, *IFN* interferons, *TERI* teriflunomide

**Fig. 4:**
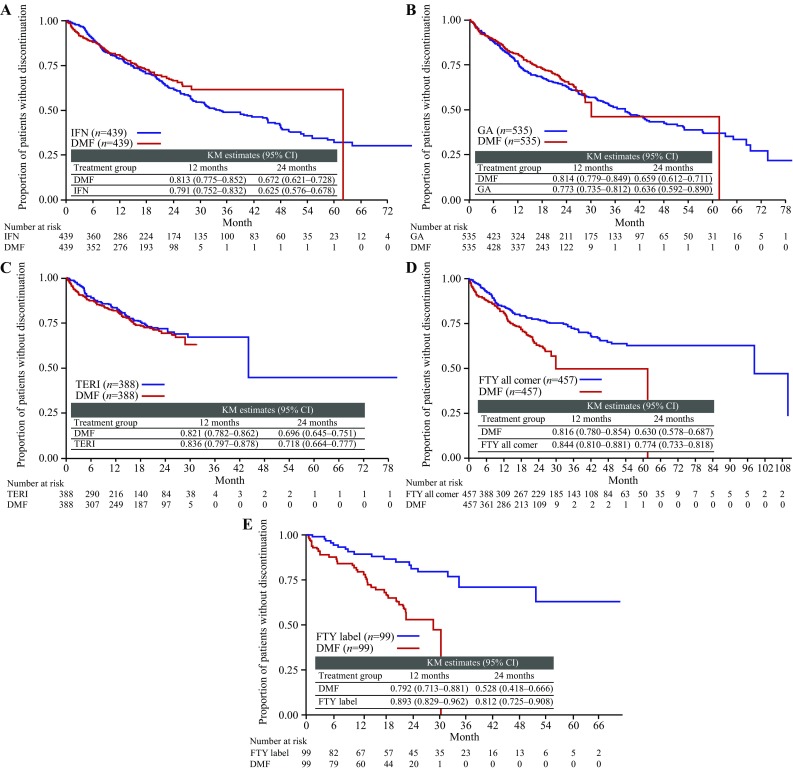
Time to index therapy discontinuation KM survival curves. **a** DMF versus IFN all-comer population; **b** DMF versus GA all-comer population; **c** DMF versus TERI all-comer population; **d** DMF versus FTY all-comer population; **e** DMF versus FTY label population. *DMF* delayed-release dimethyl fumarate, *FTY* fingolimod, *GA* glatiramer acetate, *IFN* interferons, *KM* Kaplan–Meier, *TERI* teriflunomide

By non-pairwise censoring, TTFR (primary outcome measurement) was significantly lower in the DMF (*n* = 439) versus IFN populations (*n* = 439), as shown by HR (95% CI; Fig. 1) and also indicated by the Kaplan–Meier survival curves (Fig. 2). In terms of secondary outcome measurements, a higher proportion (95% CI) of patients remained relapse free in the DMF population at 12 months [DMF 83.5% (79.7–87.6), IFN 76.5% (72.2–81.0)] and 24 months [DMF 77.0% (72.2–82.1), IFN 61.0% (55.5–67.1)]. Further, ARR (95% CI) for the DMF population [0.173 (0.137–0.219)] was significantly lower than that of the IFN population [0.245 (0.209–0.287)], resulting in an RR (95% CI) of 0.706 (0.530–0.939, *p* = 0.017; Fig. 3). TTD showed no evidence of difference between the DMF and IFN populations [HR 0.884 (95% CI 0.703–1.112), *p* = 0.293; Fig. 4]. Adverse events (AEs) and patient decision for non-medical reasons were the main reasons for discontinuation in both DMF and IFN populations, with lack of efficacy cited more commonly as a reason in the IFN population (Table 3). In terms of the exploratory analysis, times to 3- and 6-month CDP events were slightly longer and proportion of progression-free patients at 12 months was slightly higher in the DMF versus IFN populations (Supplementary Figs. 3, 4, 5).

**Table 3:** Reasons for discontinuation according to therapy

Reason for discontinuation	DMF (*n* = 439)	IFN (*n* = 439)	DMF (*n* = 535)	GA (*n* = 535)	DMF (*n* = 388)	TERI (*n* = 388)	DMF (*n* = 457)	FTY all-comer (*n* = 457)	DMF (*n* = 99)	FTY label (*n* = 99)
Total number of discontinuations	117	193	150	226	100	83	136	115	37	19
Adverse event	47 (40.2%)	54 (28.0%)	57 (38.0%)	49 (21.7%)	49 (49.0%)	43 (51.8%)	57 (41.9%)	28 (24.3%)	10 (27.0%)	6 (31.6%)
Fatigue	0 (0.0%)	2 (1.0%)	0 (0.0%)	1 (0.4%)	–	–	–	–	–	–
Lack of efficacy	13 (11.1%)	40 (20.7%)	23 (15.3%)	44 (19.5%)	6 (6.0%)	18 (21.7%)	21 (15.4%)	14 (12.2%)	11 (29.7%)	4 (21.1%)
Non-medical (patient decision)	43 (36.8%)	86 (44.6%)	51 (34.0%)	104 (46.0%)	34 (34.0%)	18 (21.7%)	44 (32.4%)	57 (49.6%)	11 (29.7%)	4 (21.1%)
Pregnancy or planned pregnancy	6 (5.1%)	8 (4.1%)	12 (8.0%)	19 (8.4%)	4 (4.0%)	3 (3.6%)	6 (4.4%)	12 (10.4%)	4 (10.8%)	5 (26.3%)
Freedom from disease activity	–	–	–	–	–	–	0 (0.0%)	1 (0.9%)	–	–
Missing	8 (6.8%)	3 (1.6%)	7 (4.7%)	9 (4.0%)	7 (7.0%)	1 (1.2%)	8 (5.9%)	3 (2.6%)	1 (2.7%)	0 (0.0%)

Data are number (proportion out of total discontinuations, %) per therapy

*DMF* delayed-release dimethyl fumarate, *FTY* fingolimod, *GA* glatiramer acetate, *IFN* interferons, *TERI* teriflunomide

### DMF versus GA

By non-pairwise censoring, TTFR (primary outcome measurement) was significantly lower in the DMF (*n* = 535) versus GA populations (*n* = 535; Fig. 1), as shown by HR (95% CI; Fig. 1) and also indicated by the Kaplan–Meier survival curves (Fig. 2). In terms of secondary outcome measurements, a higher proportion (95% CI) of patients remained relapse free in the DMF population at 12 months [DMF 82.7% (79.2–86.4), GA 75.3% (71.2–79.5)] and 24 months [DMF 76.3% (71.9–80.9), GA 64.3% (59.3–69.6)]. Further, ARR (95% CI) for the DMF population [0.187 (0.151–0.231)] was significantly lower than that of the GA population [0.246 (0.211–0.288)], resulting in an RR (95% CI) of 0.760 (0.589–0.981, *p* = 0.035; Fig. 3) in favour of DMF. TTD showed no evidence of difference in the DMF and GA populations [HR 0.926 (95% CI 0.746–1.150), *p* = 0.4881; Fig. 4]. AEs and patient decision for non-medical reasons were the main reasons for discontinuation in both the DMF and GA populations (Table 3). In terms of the exploratory analysis, time to 3- and 6-month CDP events and proportion of progression-free patients at 12 months were similar in the DMF versus GA populations (Supplementary Figs. 3, 4, 5).

### DMF versus TERI

By non-pairwise censoring, TTFR (primary outcome measurement) was significantly lower in the DMF (*n* = 388) versus TERI populations (*n* = 388), as shown by HR (95% CI; Fig. 1) and also indicated by the Kaplan–Meier survival curves (Fig. 2). In terms of secondary outcome measurements, a higher proportion (95% CI) of patients remained relapse free in the DMF population at 12 months [DMF 86.8% (83.0–90.8), TERI 79.0% (74.4–83.9)] and 24 months [DMF 81.4% (76.6–86.5), TERI 69.1% (63.2–75.5)]. Further, ARR (95% CI) for the DMF population [0.117 (0.088–0.156)] was significantly lower than that of the TERI population [0.215 (0.174–0.266)], resulting in an RR (95% CI) of 0.546 (0.387–0.771, *p* = 0.001; Fig. 3). TTD showed no evidence of difference between the DMF and TERI populations [HR 1.124 (95% CI 0.834–1.515), *p* = 0.4435; Fig. 4]. AEs and patient decision for non-medical reasons were the main reasons for discontinuation in both the DMF and TERI populations, with lack of efficacy cited more commonly as a reason in the TERI population (Table 3). In terms of the exploratory analysis, time to 3- and 6-month CDP events and proportion of progression-free patients at 12 months were similar in the DMF versus TERI populations (Supplementary Figs. 3, 4, 5).

### DMF versus FTY

By non-pairwise censoring, TTFR (primary outcome measurement) showed no evidence of significant difference between the DMF (*n* = 457) and FTY populations (*n* = 457), as shown by HR (95% CI; Fig. 1) and Kaplan–Meier survival curves (Fig. 2). In terms of secondary outcome measurements, similar proportions (95% CI) of patients remained relapse free in each treatment group at 12 months [DMF 83.6% (79.8–87.6), FTY 81.3% (77.5–85.3)]. Further, ARR (95% CI) for the DMF population [0.185 (0.148–0.232)] was similar to that of the FTY population [0.177 (0.146–0.214)], resulting in an RR (95% CI) of 1.047 (0.781–1.404, *p* = 0.759; Fig. 3). TTD was significantly longer in the FTY (115 events, 25.2%) versus DMF populations [136 events, 29.8%; HR 1.761 (1.344–2.307), *p* < 0.0001; Fig. 4]. AEs and patient decision for non-medical reasons were the main reasons for discontinuation in the DMF and FTY populations, although the proportion of patients with AEs was higher in the DMF versus FTY populations (Table 3). In terms of the exploratory analysis, time to 3- and 6-month CDP events and proportion of progression-free patients at 12 months were similar in the DMF versus FTY populations (Supplementary Figs. 3, 4, 5).

By applying the more restrictive definition of the European label the FTY sample size was reduced from 457 patients to 99 patients. There was also no evidence of difference in TTFR in terms of HR and Kaplan–Meier survival curves between the DMF and FTY label populations (Figs. 1, 2). As with the FTY all-comer population, similar proportions (95% CI) of patients remained relapse free in each treatment group at 12 months [DMF 74.3% (65.0–84.9), FTY label 72.8% (63.8–83.1)]. ARR for the DMF population also was similar to that of the FTY label population, resulting in an RR (95% CI) of 1.166 (0.707–1.922, *p* = 0.549; Fig. 3). In concordance with the results of the FTY all-comer population, TTD was significantly longer in the FTY label versus DMF populations [HR 3.305 (1.751–6.238), *p* = 0.0002; Fig. 4]. AEs, lack of efficacy, and patient decision for non-medical reasons were the main reasons for discontinuation in both the DMF and FTY label populations; pregnancy or planned pregnancy also was a common reason in the FTY label population (Table 3). In terms of the exploratory analysis, time to 3- and 6-month CDP events were slightly fewer and proportion of progression-free patients at 12 months was slightly higher in the DMF versus FTY label populations (Supplementary Figs. 3, 4, 5).

### Sensitivity analysis by pairwise censoring analysis

For each treatment comparison with DMF, results obtained by pairwise censoring analysis were consistent with those obtained by non-pairwise censoring analysis (Figs. 1, 2, Supplementary Fig. 5). For pairwise censoring analyses, the proportion of patients with relapses per category (1, 2, 3, 4+) were lower in the DMF versus IFN, GA, TERI, and FTY all-comer populations, and were similar between the DMF and FTY label populations (Supplementary Table 4).

## Discussion

Despite the primary role of RCTs for establishing efficacy and safety of new interventions or treatments compared with placebo or active control, several inherent shortcomings make their results difficult to generalise to real-world practice. Firstly, RCTs are unable to provide comparative information regarding all available treatment options. Secondly, patients enrolled in RCTs are selected against strict criteria and thereby do not reflect the broad range of patient characteristics, treatment history, comorbidities, and other factors seen in real-world cohorts. Moreover, patients, doctors, and payers now expect more reliable and transparent information to guide treatment decisions and resource allocation. National and multinational MS registries enable high-quality data acquisition based on modern technology. Advanced statistical methods such as PSM to ensure comparability of the treatment cohorts provide a scientifically sound and statistically rigorous basis for robust results to support the shared decision process between doctors and patients when selecting DMTs in daily practice.

This analysis of the German NTD MS registry implemented best practice in the analysis of non-randomised studies and real-world data to minimise the risk of the most critical bias in the following way [21–23]. (A) PSM methods to minimise the risk of  a selection bias when comparing matched patients treated with DMF and comparator DMTs [24]. It should be noted as for any other PS-based analysis, the PS are only based on measured confounders and cannot account for unmeasured confounders, for example MRI or cognition could potentially be unmeasured confounders for this study. No evidence of significance difference between DMF versus FTY was detected in a sensitivity analysis (Supplementary Table 6). (B) Clear and unique relapse definition and certified raters in the EDSS assessment across NTD to minimise the risk of a detection bias, which may arise if the outcome measurement of interest (either relapse or EDSS in this study) is differently assessed between cohorts. (C) The approximately 3-monthly visit schedule (including relapse and EDSS assessment) across all cohorts (DMF and comparator cohorts) may mitigate the risk of a performance bias. Detailed information on median follow-up times is located in Supplementary Table 5. (D) A sensitivity analysis based on pairwise censoring was implemented to account for different follow-up time between the cohorts and therefore to mitigate the risk of attrition bias.

In this study, DMF therapy following previous relapse either on or off therapy proved to be superior to IFN, GA, and TERI regarding relapse activity. This is in line with results from other studies. Patients with high disease activity require a different perspective and, among the DMTs under investigation in this study, only FTY is labelled specifically for the treatment of such patients. In this study, DMF showed no evidence of significant difference with FTY in relapse outcome measurements. Importantly, this study had both a relatively large sample size and lengthy and frequent follow-up, while previous reports often have only large sample size or extensive, high-quality follow-up, but not both [3, 7, 10–14]. Median follow-up frequency between visits, including EDSS assessment within NTD, is approximately 3 months. Of particular importance is the subgroup analysis demonstrating that the high efficacy of DMF, similar to FTY, also is seen in patients with RRMS with high disease activity and previous DMT failure. The results of our primary analysis extend those of previous studies, reinforcing the greater effectiveness of DMF relative to IFN, GA, and TERI, and similar effectiveness to FTY; however, our study also employed a sensitivity analysis using pairwise censoring to show the robustness of the results. This sensitivity analysis, which accounts for differences in treatment follow-up time, yielded results that were consistent with the primary non-censored analysis across all comparisons, supporting the robustness of these results.

TTD was found to be similar between DMF and IFN, GA, or TERI, while patients treated with FTY had a longer TTD. This suggests that robust and comparable patient adherence can be achieved with DMF if recommendations to mitigate gastrointestinal AEs during initiation of DMF are followed routinely, as they are throughout the NTD network. Such recommendations include patient coaching, taking DMF with food, slow dose titration, dose modification, and use of symptomatic therapies. Other common reasons for discontinuation seen consistently throughout treatment populations were patient decision for non-medical reasons and lack of efficacy.

We expect that longer-term observation will provide additional results and clarify the relative effect of DMF and comparators on disability progression in the future.

Previous CE studies have compared effectiveness of DMF with other DMTs, including direct and indirect comparisons of clinical trial or real-world data, some having incorporated PSM analysis methods. Overall, this PSM registry analysis is well supported by findings of previous CE studies. Data from clinical trials using comparisons based on post hoc direct, mixed treatment, or matching-adjusted indirect methods consistently support the results of this analysis, especially in terms of ARR absolute values and RRs [1–5]. For example, the ARR RRs for DMF versus IFN, GA, and TERI noted in this real-world evidence from PSM data are highly consistent with those noted in direct and indirect comparisons of clinical trial data [1, 5]. Following clinical trial data analysis, real-world evidence from analysis of insurance claims databases and patient data from academic medical centres emerged [7, 8, 10–13]. Studies from these sources also found that treatment with DMF provided efficacy/effectiveness greater than IFN, GA, or TERI, and similar to that of FTY regardless of whether the comparison method was direct or involved propensity-adjusted cohorts. For example, a retrospective analysis of US claims data found that ARRs for DMF were lower than those for IFN, GA, and TERI and similar to those for FTY [7]. Finally, real-world evidence has progressed to studies based on data from large multinational registries, including this analysis of the NTD registry [14]. A previous PSM analysis of the MSBase registry (http://www.msbase.org) closely reflects this NTD registry analysis with respect to both methods, as well as TTFR, ARR, and discontinuation results [14].

Although the existing body of evidence that supports CE of DMF is generally based on well-conducted studies, this PSM analysis of NTD registry data can be differentiated from previous studies in several respects, including data sources and method of analysis, cohort types, and the nature of results (e.g. outcome measurements considered, length of observation or follow-up). The present PSM analysis is based on routinely collected data from outpatients seen in clinical practice and under real-world treatment conditions. The clinical practice data follow-up frequency was approximately every 3 months. This contrasts with clinical trial data, which may not properly represent real-world conditions. Insurance claims databases can provide real-world data but, unlike the current PSM analysis, generally give limited information on diagnostic criteria, disease severity, rate of progression, or EDSS status. Data from retrospective studies (ie, chart review) of patients from academic medical centres can provide such clinical data, but patient numbers are often less robust than with claims or registry data sources. Regarding cohort types, the present PSM analysis is based on outpatients seen in routine clinical practice and, to our knowledge, is the first to include patients who meet the more stringent requirements of the European label for FTY. In terms of methodology, this analysis employed best-practice PSM methods and confirming the robustness of results based on applied sensitivity analyses. In terms of specific results, the present PSM analysis includes a greater number of outcome measurements than typically used in previous studies. For example, data on discontinuation has not been consistently included in previous real-world effectiveness studies or post hoc analyses of clinical trials [1, 5, 7, 8, 13]. Finally, this PSM analysis includes results based on up to 2 years of observation, whereas results from previous studies are often based on observation periods of up to only 1 year in duration [7, 11, 12]. Hence, while most CE studies have been well conducted according to certain criteria, the present PSM analysis of NTD registry data is distinct in fulfilling these criteria most comprehensively.

Following previous relapse either on or off therapy, DMF was superior to IFN, GA, and TERI on relapse outcome measurements; in addition, there was no evidence of significant difference in efficacy between DMF and FTY, including among patients with high disease activity who met the European criteria for FTY. These results confirm those from previous CE studies and also provide additional support based on use of state-of-the-art PSM practices, careful cohort selection, and comprehensive inclusion of outcome measurements, as well as longer observation than several previous studies. The present PSM provides useful data for clinical decision making based on patient-relevant outcome measurements and further insight into comparative efficacy of commonly used agents for RRMS in a real-world treatment setting.

## Electronic supplementary material

Below is the link to the electronic supplementary material.

Supplementary material 1 (DOCX 2144 KB)
