# Supplementary material for: Comparative effectiveness of delayed-release dimethyl fumarate versus interferon, glatiramer acetate, teriflunomide, or fingolimod: results from the German NeuroTransData registry
Source: J Neurol. 2018 Oct 16;265(12):2980–92. doi: 10.1007/s00415-018-9083-5 (PMC6244642; doi:10.1007/s00415-018-9083-5)
Supplement: Supplementary file 1 — Supplementary material 1 (DOCX 2144 KB) [file 415_2018_9083_MOESM1_ESM.docx]

Supplementary appendix

Supplement to: Braune S, et al. Comparative effectiveness of delayed-release dimethyl fumarate versus interferon, glatiramer acetate, teriflunomide, or fingolimod: results from the German NeuroTransData registry.

**Table of contents**

[Supplementary Table 1 Baseline characteristics of unmatched populations for the IFN, GA, and TERI comparison populations 2](#_Toc512844788)

[Supplementary Table 2 Baseline characteristics of unmatched populations for the DMF and FTY all-comer and FTY label comparison populations 3](#_Toc512844789)

[Supplementary Table 3 Exposure time according to non-pairwise censoring 4](#_Toc512844790)

[Supplementary Table 4 Number and proportion of patients with relapses by pairwise censoring 5](#_Toc512844791)

Supplementary Table 5 Median follow up time according to therapy 6

Supplementary Table 6 Sensitivity analysis 7

[Supplementary Fig. 1 Flowchart of patient disposition. 8](#_Toc512844792)

[Supplementary Fig. 2 Propensity score density distributions before and after matching for DMF and comparator populations. 9](#_Toc512844793)

[Supplementary Fig. 3 Time to 3-month EDSS confirmed disability progression KM curves by non-pairwise censoring. 11](#_Toc512844794)

[Supplementary Fig. 4 Time to 6-month EDSS confirmed disability progression KM curves by non-pairwise censoring. 12](#_Toc512844795)

[Supplementary Fig. 5 Time to first relapse KM curves by pairwise censoring. 13](#_Toc512844796)

# Supplementary Table 1 Baseline characteristics of unmatched populations for the IFN, GA, and TERI comparison populations

| **Baseline factor** | **Unmatched** | | | | | | | | | | | |
| --- | --- | --- | --- | --- | --- | --- | --- | --- | --- | --- | --- | --- |
|  | **DMF versus IFN** | | | | **DMF versus GA** | | | | **DMF versus TERI^a^** | | | |
|  | **DMF (*n*=35)** | **IFN (*n*=946)** | **d** | ***p*-value** | **DMF (*n*=165)** | **GA (*n*=174)** | **d** | ***p*-value** | **DMF (*n*=529)** | **TERI (*n*=0)** | **d** | **p-value** |
| Female | 74.3% | 70.4% | 0.087 | 0.759 | 86.8% | 66.1% | 0.503 | 0.000 | 76.4% |  |  |  |
| Age (years) | 49.9 (6.9) | 35.1 (9.7) | –1.728 | 0.000 | 40.9 (10.8) | 35.1 (9.5) | –0.568 | 0.000 | 36.6 (9.62) |  |  |  |
| Median EDSS (Q25, Q75) | 3  (1.5, 4) | 1  (0, 2) | –0.881 | 0.000 | 2  (1, 3) | 1.5  (0, 2) | –0.478 | 0.000 | 1.5  (1, 2.5) |  |  |  |
| Disease duration in months (SD) | 166.9 (98.0) | 46.1 (66.9) | –1.439 | 0.000 | 140.5 (106.4) | 28.7 (35.4) | –1.409 | 0.000 | 82.0 (71.7) |  |  |  |
| Prior number of DMTs, % |  |  |  |  |  |  |  |  |  |  |  |  |
| 0 | 0% | 99.3% |  |  | 1.2% | 97.7% |  |  | 35.7% |  |  |  |
| 1 | 97.1% | 0.7% | 16.381 | 0.000 | 94.5% | 2.3% | 7.356 | 0.000 | 53.1% |  |  |  |
| 2 | 2.9% | 0.0% |  |  | 4.2% | 0.0% |  |  | 11.2% |  |  |  |
| Total number of relapses in the last 12 months, % |  |  |  |  |  |  |  |  |  |  |  |  |
| 0 | 97.1% | 48.7% |  |  | 78.8% | 47.1% |  |  | 68.2% |  |  |  |
| 1 | 2.9% | 42.9% | 1.301 | 0.000 | 17.6% | 41.4% | 0.720 | 0.000 | 24.2% |  |  |  |
| 2 | 0.0% | 7.6% |  |  | 3.6% | 7.5% |  |  | 6.4% |  |  |  |
| 3+ | 0.0% | 0.7% |  |  | 0.0% | 4.0% |  |  | 1.3% |  |  |  |
|  |  |  |  |  |  |  |  |  |  |  |  |  |
| Total number of relapses in the last 24 months, % |  |  |  |  |  |  |  |  |  |  |  |  |
| 0 | 91.4% | 45.8% |  |  | 57.0% | 47.1% |  |  | 58.6% |  |  |  |
| 1 | 5.7% | 42.5% |  |  | 29.1% | 39.1% |  |  | 24.0% |  |  |  |
| 2 | 2.9% | 9.1% | 1.142 | 0.000 | 13.3% | 9.2% | 0.374 | 0.023 | 12.3% |  |  |  |
| 3 | 0.0% | 2.0% |  |  | 0% | 3.4% |  |  | 3.2% |  |  |  |
| 4+ | 0.0% | 0.6% |  |  | 0.6% | 1.1% |  |  | 1.9% |  |  |  |
| C-statistic |  | | | |  | | | |  | | | |

^a^No TERI patients were unmatched as all were propensity score matched to a patient of the respective comparator cohort

*d* standardised difference, *DMF* delayed-release dimethyl fumarate, *DMT* disease-modifying therapy, *GA* glatiramer acetate, *IFN* interferons, *EDSS* Expanded Disability Status Scale, *TERI* teriflunomide

# Supplementary Table 2 Baseline characteristics of unmatched populations for the DMF and FTY all-comer and FTY label comparison populations

| **Baseline factor** | **Unmatched** | | | | | | | |
| --- | --- | --- | --- | --- | --- | --- | --- | --- |
|  | **DMF versus FTY all comer** | | | | **DMF versus FTY label^a^** | | | |
|  | **DMF (*n*=336)** | **FTY (*n*=316)** | **d** | ***p*-value** | **DMF (*n*=0)** | **FTY (*n*=321)** | **d** | ***p*-value** |
| Female | 70.2% | 68.4% | 0.041 | 0.663 |  | 66.4% |  |  |
| Age in years (SD) | 40.7 (10.5) | 37.9 (10.4) | –0.269 | 0.000 |  | 39.7  (10.4) |  |  |
| Median EDSS  (Q25, Q75) | 1  (0, 2) | 2.5  (1.5, 4) | 1.031 | 0.000 |  | 2.5  (1.5, 4) |  |  |
| Mean disease duration in months (SD) | 89.3 (92.5) | 104.4 (80.2) | 0.175 | 0.000 |  | 114.5 (87.2) |  |  |
| Prior number of DMT, % |  |  |  |  |  |  |  |  |
| 0 | 66.7% | 1.9% |  |  |  | 85.0% |  |  |
| 1 | 25.6% | 82.3% | 1.899 | 0.000 |  | 15.0% |  |  |
| 2 | 7.1% | 15.8% |  |  |  | 0.0% |  |  |
| 3 | 0.6% | 0.0% |  |  |  | 0.0% |  |  |
| Total number of relapses in the last 12 months, % |  |  |  |  |  |  |  |  |
| 0 | 83.8% | 0.6% |  |  |  | 0.0% |  |  |
| 1 | 16.4% | 50.6% |  |  |  | 55.8% |  |  |
| 2 | 0.3% | 33.2% | 3.323 | 0.000 |  | 31.2% |  |  |
| 3 | 0.0% | 13.6% |  |  |  | 11.2% |  |  |
| 4+ | 0.0% | 1.9% |  |  |  | 1.9% |  |  |
| Total number of relapses in the last 24 months, % |  |  |  |  |  |  |  |  |
| 0 | 71.1% | 0.6% |  |  |  | 0.0% |  |  |
| 1 | 26.8% | 27.5% |  |  |  | 31.8% |  |  |
| 2 | 1.5% | 34.5% | 2.887 | 0.000 |  | 34.4% |  |  |
| 3 | 0.6% | 23.7% |  |  |  | 20.9% |  |  |
| 4+ | 0.0% | 13.6% |  |  |  | 13.1% |  |  |
| C-statistic |  | | | |  | | | |

^a^No DMF patients were unmatched as all were propensity score matched to a patient of the respective comparator cohort

*d* standardised difference, *DMF* delayed-release dimethyl fumarate, *DMT* disease-modifying therapy *EDSS* Expanded Disability Status Scale, *FTY* fingolimod

# Supplementary Table 3 Exposure time according to non-pairwise censoring

| **Index-Therapy** | **n** | **Q25** | **Median** | **Q75** |
| --- | --- | --- | --- | --- |
| DMF versus IFN | | | | |
| IFN | 439 | 7.852 | 18.924 | 33.593 |
| DMF | 439 | 7.573 | 15.967 | 23.524 |
| DMF versus GA | | | | |
| GA | 535 | 6.817 | 15.474 | 35.811 |
| DMF | 535 | 7.573 | 16.361 | 23.655 |
| DMF versus TERI | | | | |
| TERI | 388 | 5.881 | 13.405 | 22.825 |
| DMF | 388 | 7.680 | 17.528 | 24.000 |
| DMF versus FTY all comer | | | | |
| FTY | 457 | 8.608 | 24.148 | 41.101 |
| DMF | 457 | 7.622 | 16.329 | 23.721 |
| DMF versus FTY label | | | | |
| FTY | 99 | 7.556 | 22.472 | 34.398 |
| DMF | 99 | 7.014 | 15.277 | 22.637 |

*DMF* delayed-release dimethyl fumarate, *FTY* fingolimod, *GA* glatiramer acetate, *IFN* interferons, *Q25* 25th quantile, *Q75* 75th quantile, *TERI* teriflunomide

# Supplementary Table 4 Number and proportion of patients with relapses by pairwise censoring

| Index-Therapy | Relapse category | | | | |
| --- | --- | --- | --- | --- | --- |
|  | **0** | **1** | **2** | **3** | **4+** |
| **DMF (***n*=**439)** | 382 (0.870) | 47 (0.107) | 7 (0.016) | 3 (0.007) | 0 (0.000) |
| **IFN (***n*=**439)** | 350 (0.797) | 71 (0.162) | 14 (0.032) | 3 (0.007) | 1 (0.002) |
| **DMF (***n***=535)** | 465 (0.869) | 53 (0.099) | 12 (0.022) | 4 (0.007) | 1 (0.002) |
| **GA (***n***=535)** | 429 (0.802) | 81 (0.151) | 20 (0.037) | 4 (0.007) | 1 (0.002) |
| **DMF (***n***=388)** | 354 (0.912) | 31 (0.080) | 3 (0.008) | 0 (0.000) | 0 (0.000) |
| **TERI (***n***=388)** | 329 (0.848) | 54 (0.139) | 4 (0.010) | 0 (0.000) | 1 (0.003) |
| **DMF (***n***=457)** | 398 (0.871) | 45 (0.098) | 11 (0.024) | 2 (0.004) | 1 (0.002) |
| **FTY all (***n***=457)** | 383 (0.838) | 62 (0.136) | 9 (0.020) | 1 (0.002) | 2 (0.004) |
| **DMF (***n***=99)** | 78 (0.788) | 16 (0.162) | 3 (0.030) | 2 (0.020) | 0 (0.000) |
| **FTY label (***n***=99)** | 77 (0.778) | 18 (0.182) | 3 (0.030) | 1 (0.010) | 0 (0.000) |

Data are number (proportion) of patients per relapse category. *DMF* delayed-release dimethyl fumarate, *FTY* fingolimod, *GA* glatiramer acetate, *IFN* interferons, *TERI* teriflunomide,

#

**Supplementary Table 5** Follow-up frequency (based on EDSS assessment) according to index therapy

| **Variable** | **DMF**  **(*n*=401)** | **IFN**  **(n=395)** | **DMF**  **(*n*=477)** | **GA**  **(*n*=442)** | **DMF**  **(*n*=348)** | **TERI**  **(*n*=344)** | **DMF**  **(*n*=400)** | **FTY all comer**  **(*n*=419)** | **DMF**  **(*n*=86)** | **FTY label**  **(*n*=93)** |
| --- | --- | --- | --- | --- | --- | --- | --- | --- | --- | --- |
| Median months between EDSS measurements on therapy | 2.76 | 2.89 | 2.76 | 2.99 | 2.76 | 2.76 | 2.78 | 2.83 | 2.78 | 2.92 |
| Median months between EDSS measurements from therapy start to patients’ last EDSS (irrespective of index-therapy discontinuation) | 2.76 | 2.79 | 2.76 | 2.92 | 2.86 | 2.89 | 2.79 | 2.76 | 2.76 | 2.76 |

*DMF* delayed-release dimethyl fumarate, *FTY* fingolimod, *GA* glatiramer acetate, *IFN* interferons, *TERI* teriflunomide

**Supplementary Table 6**

A sensitivity analysis of an unmeasured confounder, following Lin et al. 1998[1], was conducted to assess the robustness of the results of the comparison DMF (n = 457) vs FTY (n = 457); non-pairwise censoring was applied.

The results of the sensitivity analysis found that for TTFR, the conclusion of no evidence of significant difference between DMF and FTY might be altered by the following hypothetical, and extreme case: an unmeasured confounder with an HR = 2.0 and a difference of the prevalence of the unmeasured confounder between DMF and FTY of at least 60%.

| **Sensitivity Analysis (selected scenarios only) for TTFR and ARR: How the Magnitude of an Unmeasured Binary Confounder Might Affect the Observed Hazard Ratios/ Rate Ratios*** | | | | | |
| --- | --- | --- | --- | --- | --- |
|  | **Results** | **Sensitivity Analysis** | | | |
| **Outcome measurement** | **HR (95% CI)** | **HR^a^** | **P_DMF_-P_FTY_^b^** | **HR** | **95% CI** |
| TTFR | 0.910 (0.676-1.224) | 1.5 | No realistic scenario possible | | |
|  |  | 2.0 | -0.6 | 1.37 | 1.01-1.84 |
|  |  | 2.5 | -0.4 | 1.38 | 1.03-1.86 |
|  |  | 3.0 | -0.3 | 1.37 | 1.01-1.84 |
|  | | | | | |
|  | **Results** | **Sensitivity Analysis** | | | |
| **Outcome measurement** | **RR (95% CI)** | **RR^a^** | **P_DMF_-P_FTY_^b^** | **RR** | **95% CI** |
| ARR | 1.047 (0.781-1.404) | 1.5 | -0.6 | 1.36 | 1.02-1.83 |
|  |  | 2.0 | -0.4 | 1.43 | 1.07-1.91 |
|  |  | 2.5 | -0.3 | 1.46 | 1.09-1.95 |
|  |  | 3.0 | -0.2 | 1.4 | 1.04-1.87 |
|  | | | | | |
| *This analysis assumes that (1) the unmeasured confounder is binary, (2) the unmeasured confounder is independent of measured  confounders, and (3) no interaction occurs between the unmeasured confounder and exposure.  ^a^Hypothetical HR/ RR, respectively of the unmeasured confounder on the outcome measurement TTFR/ ARR, respectively.  ^~~b~~^Hypothetical differences in the prevalence of the unmeasured confounder between DMF vs. FTY; a negative value means that the prevalence of the unmeasured confounder is higher in FTY treatment group.  *ARR* annualized relapse rate, *CI* confidence interval, *DMF* delayed-release dimethyl fumarate, *FTY* fingolimod, *HR* hazard ratio, *RR* rate ratio, *TTFR* time to first relapse | | | | | |

# Supplementary Fig. 1 Flowchart of patient disposition. Note: individual patients may have received therapies on multiple occasions. *DMF* delayed-release dimethyl fumarate, *FTY* fingolimod, *GA* glatiramer acetate, *IFN* interferons, *RRMS* relapsing-remitting multiple sclerosis, *TERI* teriflunomide


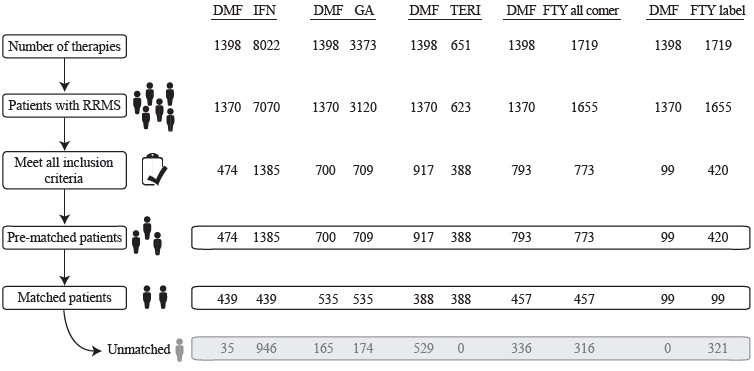


# Supplementary Fig. 2 Propensity score density distributions before and after matching for DMF and comparator populations. (A) IFN (B) GA, (C) TERI, (D) FTY all-comer, and (E) FTY label populations. *DMF* delayed-release dimethyl fumarate, *FTY* fingolimod, *GA* glatiramer acetate, *IFN* interferons, *TERI* teriflunomide


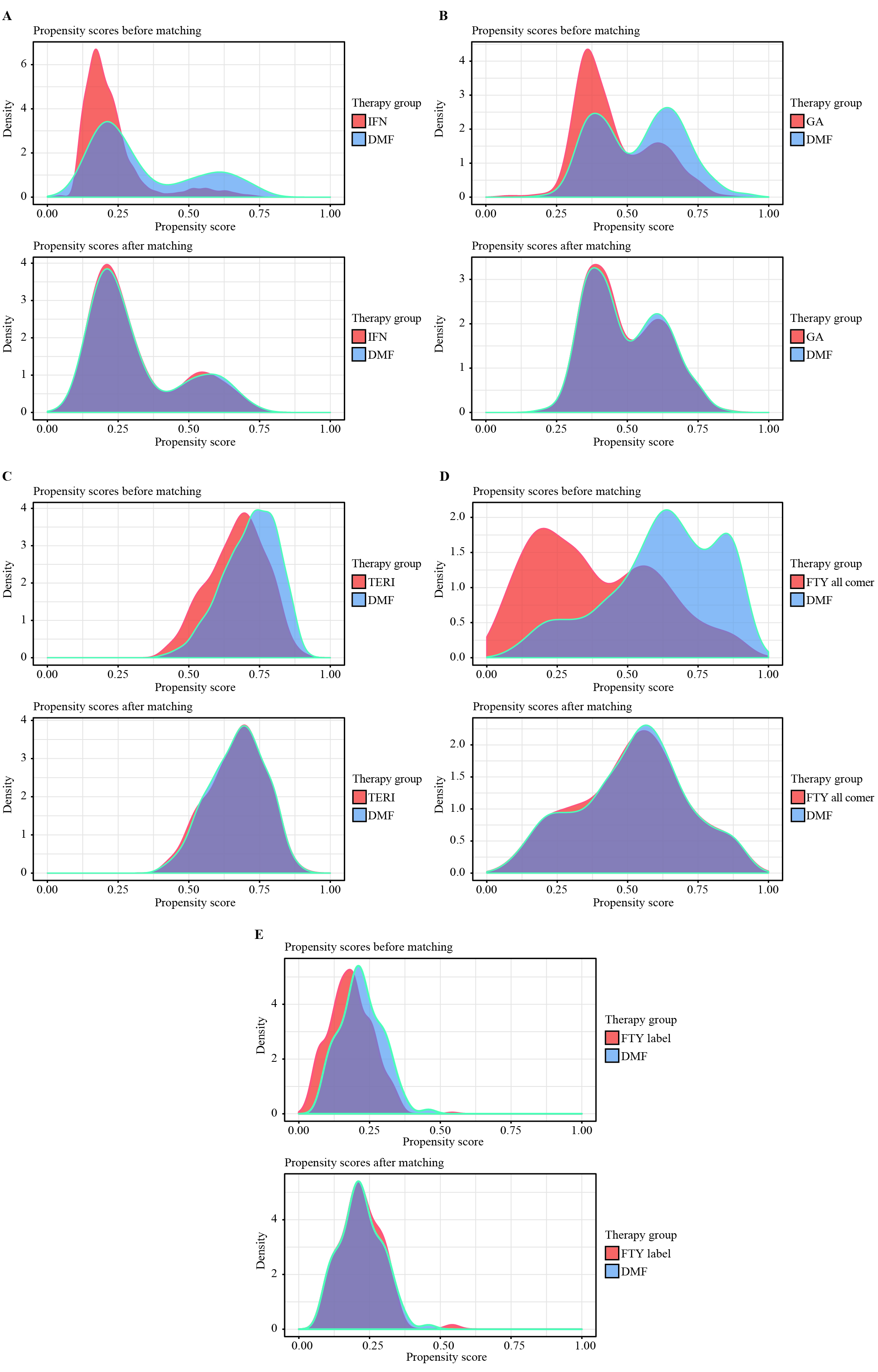


# Supplementary Fig. 3 Time to 3-month EDSS confirmed disability progression KM curves by non-pairwise censoring. (A) DMF versus IFN all-comer population, (B) DMF versus GA all-comer population, (C) DMF versus TERI all-comer population, (D) DMF versus FTY all-comer population, and (E) DMF versus FTY label population. *DMF* delayed-release dimethyl fumarate, *EDSS* Expanded Disability Status Scale, *FTY* fingolimod, *GA* glatiramer acetate, *IFN* interferons, *KM* Kaplan-Meier, *TERI* teriflunomide


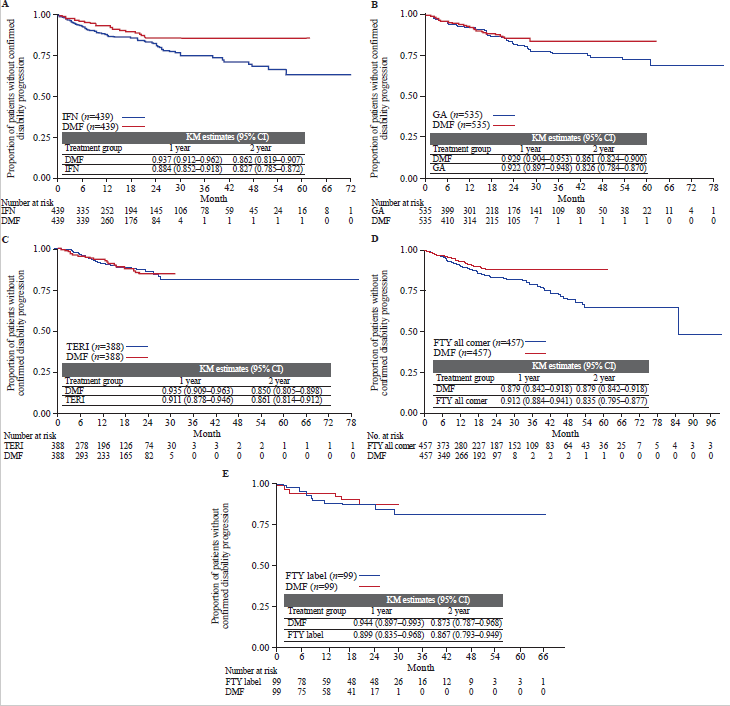


# Supplementary Fig. 4 Time to 6-month EDSS confirmed disability progression KM curves by non-pairwise censoring. (A) DMF versus IFN all-comer population, (B) DMF versus GA all-comer population, (C) DMF versus TERI all-comer population, (D) DMF versus FTY all-comer population, and (E) DMF versus FTY label population. *DMF* delayed-release dimethyl fumarate, *EDSS* Expanded Disability Status Scale, *FTY* fingolimod, *GA* glatiramer acetate, *IFN* interferons, *KM* Kaplan-Meier, *TERI* teriflunomide


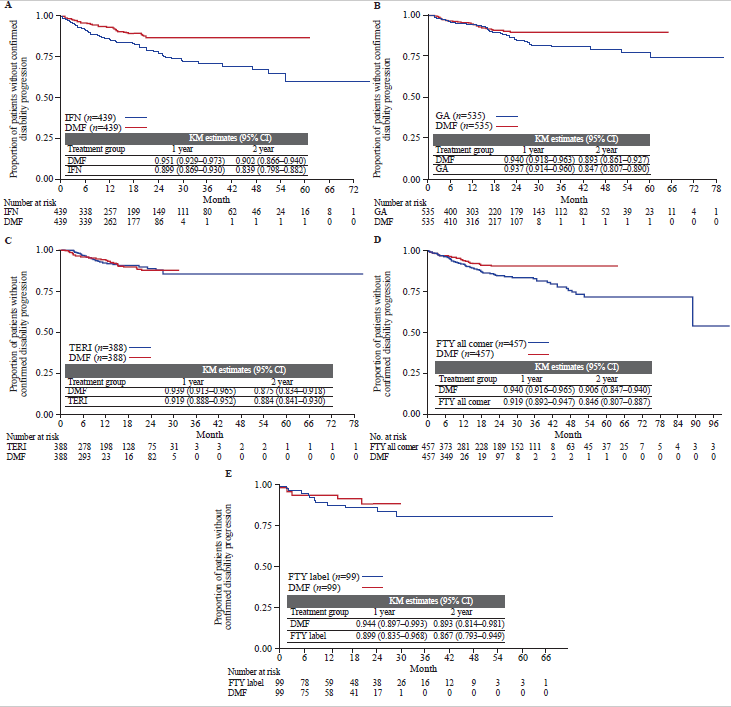


# Supplementary Fig. 5 Time to first relapse KM curves by pairwise censoring. (A) DMF versus IFN all-comer population, (B) DMF versus GA all-comer population, (C) DMF versus TERI all-comer population, (D) DMF versus FTY all-comer population, and (E) DMF versus FTY label population. *DMF* delayed-release dimethyl fumarate, *FTY* fingolimod, *GA* glatiramer acetate, *IFN* interferons, *KM* Kaplan-Meier, *TERI* teriflunomide


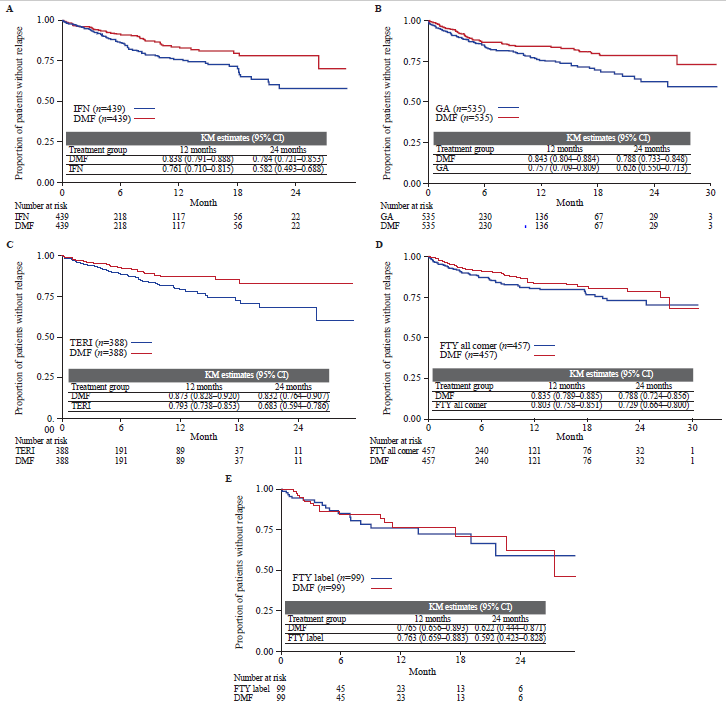


References

1. Lin DY, Psaty BM, Kronmal RA (1998) Assessing the sensitivity of regression results to unmeasured confounders in observational studies. Biometrics 54:948-963
